# Supplementary figures and images for: Seventeen-year study reveals fluctuations in key ecological indicators on two reef crests in Cuba
Source: PeerJ. 2024 Jan 23;12:e16705. doi: 10.7717/peerj.16705 (PMC10812586; doi:10.7717/peerj.16705)

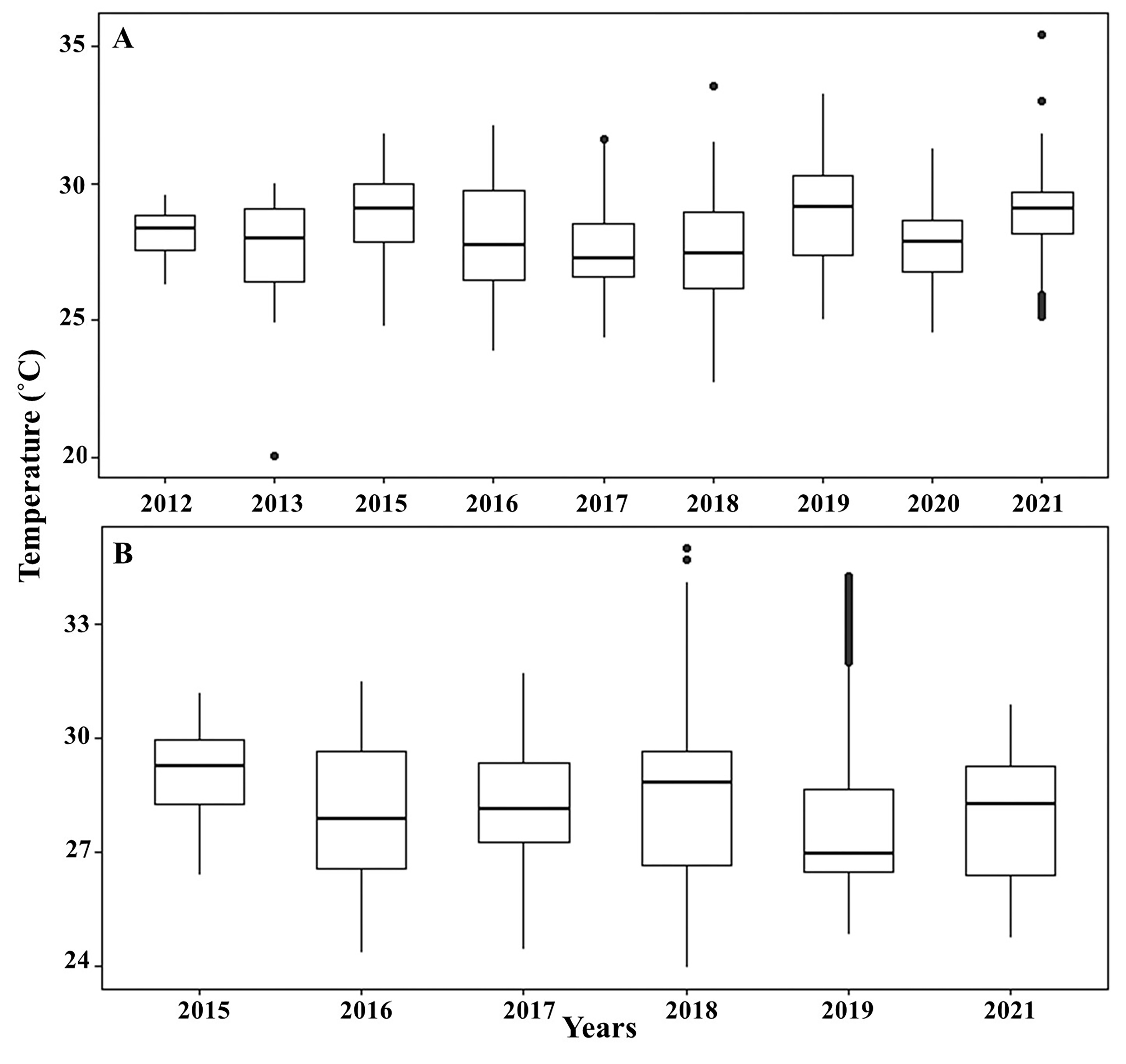

Supplement: Supplemental Information 1 — Sea water temperature at Playa Baracoa (A) and Rincon de Guanabo (B) from 2012 to 2021. The horizontal black line inside the box represents the mean, the size of the box is the interquartile range, and the whiskers represent the minimum and maximum data values. Black circles indicate outliers. [file peerj-12-16705-s001.jpg]
