# Supplementary material for: Seventeen-year study reveals fluctuations in key ecological indicators on two reef crests in Cuba
Source: PeerJ. 2024 Jan 23;12:e16705. doi: 10.7717/peerj.16705 (PMC10812586; doi:10.7717/peerj.16705)
Supplement: Supplemental Information 2 — Sample size by variables across years and site. The band transect (10 m2) was the sampling unit that was used for the determinations of Acropora palmata and Diadema antillarum density. The linear transect (10 m) was employed when determining colony diameter and health as well as benthic cover. [file peerj-12-16705-s002.docx]

| Site | Variable | Year | Sample size |
| --- | --- | --- | --- |
|  |  | 2005 | 40 |
|  |  | 2008 | 60 |
|  |  | 2012 | 50 |
| Playa Baracoa |  | 2013 | 50 |
|  |  | 2015 | 60 |
|  | *A. palmata* density | 2017 | 60 |
|  |  | 2021 | 60 |
|  |  | 2008 | 60 |
|  |  | 2015 | 60 |
| Rincon de Guanabo |  | 2017 | 60 |
|  |  | 2021 | 60 |
|  |  | 2005 | 280 |
|  |  | 2006 | 456 |
| Playa Baracoa |  | 2012 | 185 |
|  |  | 2013 | 432 |
|  | *A. palmata* diameter | 2015 | 605 |
|  |  | 2021 | 623 |
|  |  | 2006 | 456 |
| Rincon de Guanabo |  | 2015 | 600 |
|  |  | 2021 | 738 |
|  |  | 2005 | 280 |
|  |  | 2006 | 456 |
|  |  | 2012 | 192 |
| Playa Baracoa |  | 2013 | 437 |
|  |  | 2015 | 612 |
|  |  | 2016 | 600 |
|  | *A. palmata* health | 2017 | 742 |
|  |  | 2021 | 623 |
|  |  | 2006 | 456 |
|  |  | 2015 | 600 |
| Rincon de Guanabo |  | 2016 | 600 |
|  |  | 2017 | 1442 |
|  |  | 2021 | 738 |
| Playa Baracoa |  | 2015 | 60 |
|  | Benthic cover | 2021 | 60 |
| Rincon de Guanabo |  | 2015 | 60 |
|  |  | 2021 | 60 |
|  |  | 2005 | 40 |
|  |  | 2008 | 60 |
| Playa Baracoa |  | 2015 | 60 |
|  | *D. antillarum* density | 2021 | 60 |
|  |  | 2008 | 60 |
| Rincon de Guanabo |  | 2015 | 60 |
|  |  | 2021 | 60 |
