# Supplementary material for: Seventeen-year study reveals fluctuations in key ecological indicators on two reef crests in Cuba
Source: PeerJ. 2024 Jan 23;12:e16705. doi: 10.7717/peerj.16705 (PMC10812586; doi:10.7717/peerj.16705)
Supplement: Supplemental Information 3 — Analyses and R models used and the key results that were found. [file peerj-12-16705-s003.docx]

| **Variable and Models** | **Key results** |
| --- | --- |
| Density of *A. palmata*  (Data fit to a Poisson distribution)  Generalized linear model | glm(density~year, data=data, family="poisson")    post hoc: Dunn's test  **Data: density of *A. palmata* in Playa Baracoa**  Deviance Residuals:  Min 1Q Median 3Q Max  -4.6018 -2.7586 -0.8686 1.3393 11.1058  Coefficients:  Estimate Std. Error z value Pr(>\|z\|)  (Intercept) 208.721125 6.751290 30.92 <2e^-16^ ***  Density$year -0.102565 0.003357 -30.55 <2e^-16^ ***  **Data: density of *A. palmata* in Rincon de Guanabo**  Deviance Residuals:  Min 1Q Median 3Q Max  -6.1062 -2.3833 -0.5991 1.2232 9.0835  Coefficients:  Estimate Std. Error z value Pr(>\|z\|)  (Intercept) 106.670135 5.816987 18.34 <2e^-16^ ***  Density $year -0.051435 0.002888 -17.81 <2e^-16^ *** |
| Sizes of *A. palmata*  (Data fit to a Poisson distribution)  Generalized linear model | glm(density ~ year, data = data, family = "poisson")  post hoc: Dunn's test  **Data: size in Playa Baracoa**  Deviance Residuals:  Min 1Q Median 3Q Max  -1.6090 -0.8175 -0.8175 -0.4576 4.8455  Coefficients:  Estimate Std. Error z value Pr(>\|z\|)  (Intercept) 388.72588 50.72999 7.663 1.82e^-14^ ***  size$year -0.19346 0.02524 -7.666 1.78e^-14^ ***  Null deviance: 407.52 on 179 degrees of freedom  Residual deviance: 325.62 on 178 degrees of freedom  AIC: 422.19  Number of Fisher Scoring iterations: 6  **Data: size in Rincon de Guanabo**  Deviance Residuals:  Min 1Q Median 3Q Max  -8.5867 -4.8709 -2.7810 0.6591 31.3619  Coefficients:  Estimate Std. Error z value Pr(>\|z\|)  (Intercept) 59.4881770 1.3508546 44.04 <2e^-16^ ***  size$year -0.0277967 0.0006707 -41.45 <2e^-16^ ***  (Dispersion parameter for poisson family taken to be 1)  Null deviance: 66312 on 1793 degrees of freedom  Residual deviance: 64628 on 1792 degrees of freedom  AIC: Inf  Number of Fisher Scoring iterations: 5 |
| density of *Diadema*  (-Data fit to a normal distribution after transformation, in Playa Baracoa.  -Data fit to a Poisson distribution in Rincon de Guanabo)  Generalized linear model | **Data: density of *Diadema* in Playa Baracoa**  lm(density ~ year, data=data)  post hoc: Tukey test  Residuals:  Min 1Q Median 3Q Max  -3.9774 -0.8690 0.0258 0.9216 4.3275  Coefficients:  Estimate Std. Error t value Pr(>\|t\|)  (Intercept) 106.38095 32.45507 3.278 0.00122 **  Año -0.05082 0.01612 -3.152 0.00185 **  Residual standard error: 1.455 on 218 degrees of freedom  Multiple R-squared: 0.04359, Adjusted R-squared: 0.0392  F-statistic: 9.935 on 1 and 218 DF, p-value: 0.00185  **Data: density of *Diadema* in Rincon de Guanabo**  glm(formula =density ~ year,  family = "poisson", data =data)  post hoc: Dunn's test  Deviance Residuals:  Min 1Q Median 3Q Max  -1.6090 -0.8175 -0.8175 -0.4576 4.8455  Coefficients:  Estimate Std. Error z value Pr(>\|z\|)  (Intercept) 388.72588 50.72999 7.663 1.82e^-14^ ***  density$year -0.19346 0.02524 -7.666 1.78e^-14^ *** |
| *D. antillarum* ~ macroalgal  [Generalized Linear Model (beta regression)](https://www.rdocumentation.org/packages/MASS/versions/7.3-58.3/topics/glm.nb) | betareg(macroalgal ~ density, data = data)  Standardized weighted residuals 2:  Min 1Q Median 3Q Max  -2.0302 -0.7377 0.1618 0.7594 2.2498  Coefficients (mean model with logit link):  Estimate Std. Error z value Pr(>\|z\|)  (Intercept) 0.538312 0.148756 3.619 0.000296 ***  Density 0.005897 0.007105 0.830 0.406538  Phi coefficients (precision model with identity link):  Estimate Std. Error z value Pr(>\|z\|)  (phi) 3.1682 0.3685 8.597 <2e^-16^ ***  Type of estimator: ML (maximum likelihood)  Log-likelihood: 21.53 on 3 Df  Pseudo R-squared: 0.007496  Number of iterations: 12 (BFGS) + 1 (Fisher scoring) |
| Benthic cover and differences between the two reefs. | Mann-Whitney U- nonparametric test |
